# Natural Tremella Polysaccharide Mitigates DEHP-Induced Oxidative Stress and Apoptosis via Dual Regulation of Survival and Antioxidant Pathways

**DOI:** 10.3390/foods14213765

**Published:** 2025-11-03

**Authors:** Xinyang Zhang, Siyuan Luo, Chengwu Cao, Tianjie Zhou, Qian He, Zhuoran Tang, Zhipeng Xie, Fengxian Liu, Dandan Wen, Hui Zou, Junnan Li

**Affiliations:** 1 Key Laboratory of Study and Discovery of Small Targeted Molecules of Hunan Province, School of Pharmacy, Health Science Center, Hunan Normal University, Changsha 410013, Chinazhoutianjie@hunnu.edu.cn (T.Z.); 202230192102@hunnu.edu.cn (Q.H.);; 2 Department of Biomedical Sciences, Faculty of Health Sciences, University of Macau, Macau SAR 999078, China

**Keywords:** Di-(2-ethylhexyl) phthalate, *Tremella fuciformis*, polysaccharides, *C. elegans*, antioxidant ability

## Abstract

Diethylhexyl phthalate (DEHP), a common environmental plasticizer, induces oxidative damage and cell apoptosis without efficient treatment. *Tremella fuciformis* polysaccharides (TFPs) are known natural antioxidants, yet their protection against DEHP toxicity remains unclear. This study aimed to investigate the protective effects of TFP against DEHP-induced toxicity using both human umbilical vein endothelial cells (HUVECs) and *Caenorhabditis elegans* models. The results demonstrate that TFPs significantly alleviated DEHP-induced cytotoxicity in HUVECs by reducing reactive oxygen species (ROS) generation and inhibiting mitochondrial apoptosis pathways, which may contribute to the activation of antioxidant systems mediating via Nrf-2. In *C. elegans*, TFP improved survival rates under DEHP stress and reduced ROS accumulation. This protection was associated with the modulation of the insulin-like pathway and *skn-1* gene to increase the expressions of antioxidant genes. Our findings reveal that TFP exhibits protection against DEHP-induced oxidative stress and apoptosis through the synergistic regulation of survival and antioxidant pathways, highlighting its potential as a natural dietary intervention for environmental toxicant-induced health risks.

## 1. Introduction

Di-(2-ethylhexyl) phthalate (DEHP), a high-molecular-weight phthalate ester derivative, is one of the most widely used plasticizers in the world. Currently, over 18 billion pounds of DEHP are used annually worldwide [1]. Its applications are widespread, spanning across numerous fields. In food and pharmaceutical packaging, DEHP enhances the flexibility and durability of materials, ensuring product stability during storage and transportation [2]. It is also crucial in processing polyvinyl chloride (PVC) and other building materials in the construction industry, improving their plasticity and processability to meet various architectural needs [3]. However, this extensive use leads to ubiquitous environmental contamination, increasing the risk of human and wildlife exposure through oral ingestion, dermal contact, and inhalation [3]. As a ubiquitous environmental endocrine disruptor, DEHP can enter the body through oral ingestion, skin contact, and injection, posing lifelong exposure risks [1,4,5]. Research has shown that DEHP exposure can lead to endocrine disruption, neurotoxicity, and reproductive toxicity [6,7]. For instance, it may cause miscarriage, abnormal sperm counts, and testicular damage and increase the risk of cancer and teratogenic effects [6,7]. The toxic mechanisms of DEHP involve its ability to inhibit cell viability in a dose-dependent manner, inducing neuronal apoptosis and increasing caspase-3 expression [8]. Moreover, DEHP activates peroxisome proliferator-activated receptors (PPARs), leading to an increase in reactive oxygen species (ROS) and other free radicals, causing oxidative stress and severe oxidative damage when the ROS clearance system’s balance is disrupted [9,10]. Given its widespread use and potential health risks, there is a pressing need to explore methods to mitigate DEHP toxicity and enhance safety.

*Tremella fuciformis*, commonly known as silver ear or snow fungus, is a large edible fungus widely cultivated in subtropical regions of China [11]. *Tremella fuciformis* polysaccharides (TFPs), the primary bioactive component of *Tremella fuciformis*, are high-molecular-weight polysaccharides mainly composed of an α-D-mannose backbone with branches of β-D-xylose, β-D-gluconic acid, and β-D-xylobiose [12]. TFP has been widely used in the food industry due to its ability to improve the gelation, stability, and emulsifying properties of food products [13]. Furthermore, it exhibits various bioactivities, including antioxidant, anti-tumor, immune regulation, anti-aging, memory improvement, anti-inflammation, and blood sugar- and lipid-lowering effects [14,15,16]. Notably, its potent antioxidant capacity suggests potential efficacy in counteracting oxidative stress-induced damage, such as that caused by DEHP [17,18]. However, while the general antioxidant properties of TFP are recognized, its specific protective mechanisms against DEHP-induced toxicity, particularly whether it relieves the oxidative stress induced by DEHP, remain poorly understood. Hence, we hypothesized that TFP can alleviate the damage induced by DEHP by exerting an antioxidant capacity.

Human umbilical vein endothelial cells (HUVECs) were selected as a relevant mammalian cell model because endothelial cells are critical targets of environmental toxicants and play a key role in vascular-related toxicity, which has been implicated in the systemic effects of DEHP [19]. The *Caenorhabditis* *elegans* model offers distinct advantages, including a well-defined genetic background, conserved stress response and apoptosis pathways, and the ability to conduct in vivo survival and genetic analyses [20]. Therefore, in this present work, HUVECs and *C. elegans* were applied to comprehensively elucidate the protective mechanisms of TFP against DEHP-induced toxicity in vitro and in vivo. The findings of this work may help to provide a novel insight for TFP to be a natural dietary intervention for reducing the health risks associated with exposure to environmental pollutants such as DEHP.

## 2. Materials and Methods

### 2.1. Cell Culture

HUVECs were maintained in DMEM supplemented with 10% fetal bovine serum and 1% penicillin–streptomycin. They were incubated at 37 °C in a humidified atmosphere of 5% CO_2_. When the cells reached 80% confluence, they were detached using trypsin–EDTA and collected for subsequent experiments.

### 2.2. Cell Viability Assay

Adjust the cell concentration to 1 × 10^5^ cells/mL. Seed cells in 96-well plates at 7000 cells per well. Incubate the plates overnight at 37 °C in a 5% CO_2_ humidified incubator. Subsequently, add polysaccharide solutions at various concentrations, with PBS as a control. After 24 h of incubation, expose the cells to a 2 mM DEHP environment for an additional 24 h. Finally, assess cell viability using the MTT method.

### 2.3. Apoptosis Analysis

We seeded the cell suspension into a 6-well plate and incubated overnight. Then w treated the cells with the polysaccharide solution for 24 h, followed by exposure to 2 mM DEHP for an additional 24 h. Then the cells were collected and washed twice with cold PBS. Subsequently, the cells were resuspended in 1× binding buffer, and Annexin V Conjugates (Thermo Fisher Scientific Inc., Waltham, MA, USA, cat: A35110) were added in for 10 min in the dark. Finally, PI solution (0.1 mg/mL) was added in for another 5 min incubation before being analyzed for apoptosis using BD flow cytometry.

### 2.4. Intracellular ROS and MMP Determination

The cell suspension was seeded into a 12-well plate and incubated overnight. Then the cells were treated with the polysaccharide solution for 24 h, followed by exposure to 2 mM DEHP for an additional 24 h. PBS was used to wash the cells twice; then ROS detection solution (Beyotime, Shanghai, China, cat: S0033M) and Rd123 solution (Beyotime, cat: C2008S) were added in, respectively. The plate was incubated in the dark for 20 min and washed three times with PBS, and we observed the intracellular ROS content and MMP level under a fluorescence microscope.

### 2.5. CAT and SOD Activity Determination

Inoculate the cell suspension into a 6 cm culture dish. Treat it with the polysaccharide solution for 24 h, and then expose it to DEHP (2 mM) for an additional 24 h. Collect and rapidly freeze the cells in liquid nitrogen. Homogenize the cells with a glass homogenizer and lyse with IP lysis buffer (Beyotime, cat: P0013). After centrifugation, collect the supernatant and measure the protein content (Beyotime, cat: P0010), CAT activity (Beyotime, cat: S0051), and SOD activity (Beyotime, cat: S0101M) following the respective protocol.

### 2.6. Western Blotting Analysis of Protein Expression

Cells were inoculated into 6 cm culture dishes, treated with polysaccharides for 24 h, and subsequently exposed to DEHP (2 mM) for another 24 h. The cells were then washed three times with PBS, collected, and lysed using SDS lysis buffer to extract protein samples. The proteins were separated by SDS-PAGE and transferred onto an NC membrane, which was blocked with 5% skimmed milk. The membrane was incubated with primary antibodies overnight at 4 °C, followed by three washes with TBST. After incubation with secondary antibodies at room temperature for 1 h and additional three TBST washes, protein bands were visualized using the ChemiDoc MP Imaging System (Bio-Rad, Hercules, CA, USA). The semi-quantitative analysis of the protein bands was performed with ImageJ (version 1.54k).

### 2.7. C. elegans Culture

The N2 wide type, CL2166 (GST-4::GFP), and *Escherichia coli* OP50 strains were obtained from the *Caenorhabditis* Genetics Center (CGC) in the United States. Nematodes were cultivated on NGM (Nematode Growth Medium) at 20 °C and fed with OP50 bacteria. Synchronized nematodes were generated using the bleach method.

### 2.8. Determination of Survival Rate

Synchronized L1-stage *C. elegans* were seeded in 96-well plates. They were treated with varying concentrations of polysaccharides for 48 h, followed by exposure to a DEHP solution at 40 mg/L for 24, 48, and 72 h. Finally, the survival rate of the nematodes was recorded.

### 2.9. Determination of ROS in Nematodes

L1-stage *C. elegans* were treated with a polysaccharide solution for 48 h and then exposed to DEHP at a concentration of 40 mg/L for 12 h. The nematodes were collected and washed using K solution. Subsequently, they were stained with an ROS detection kit (Beyotime, cat: S0033M) for 1 h. After another wash with K solution, the nematodes were transferred to 96-well plates, with 13–15 nematodes per well. Finally, the fluorescence values were measured using a microplate reader to determine the level of ROS in the nematodes.

### 2.10. Gene Expression Analysis

Synchronized L1-stage *C. elegans* were treated with the polysaccharide solution for 48 h and then exposed to DEHP (40 mg/L) for 12 h. The nematodes were collected, washed with K solution, and rapidly frozen in liquid nitrogen before being ground into a fine powder. Total RNA was extracted using an RNA extraction kit (Beyotime, cat: R0027) and reverse transcribed into cDNA (Bio-rad, cat: 1708891). Gene expression levels were quantified using the SYBR Green method.

### 2.11. Detection of CAT, SOD, and GSH Activities in Nematodes

L1-stage *C. elegans* were treated with the polysaccharide solution for 48 h and then exposed to DEHP (40 mg/L) for 12 h. The nematodes were collected, washed with K solution, and rapidly frozen in liquid nitrogen before being ground in a glass homogenizer. IP lysis buffer (Beyotime, cat: P0013) was used to assist in lysis. The resulting protein solution was centrifuged, and the supernatant was collected for the determination of protein content (Beyotime, cat: P0010), CAT activity (Beyotime, cat: S0051), SOD activity (Beyotime, cat: S0101M), and GSH activity (Beyotime, cat: S0053).

### 2.12. Visualization of GST-4::GFP

L1-stage CL2166 worms were treated with TFP for 48 h. Then the worms were exposed to DEHP for 12 h. The worms were anesthetized and observed under a fluorescence microscope.

### 2.13. Statistical Analysis

Data are presented as mean ± standard deviation (SD). Statistical comparisons between control and experimental groups were performed using GraphPad Prism 8 software. Significance levels are indicated as follows: ns for non-significant, * for *p* < 0.05, ** for *p* < 0.01, and *** for *p* < 0.001. These levels were determined using one-way or two-way ANOVA followed by Tukey’s multiple comparisons test or Student’s *t*-test as appropriate.

## 3. Results

### 3.1. TFP Alleviated DEHP-Induced Cytotoxicity

**Figure 1:**
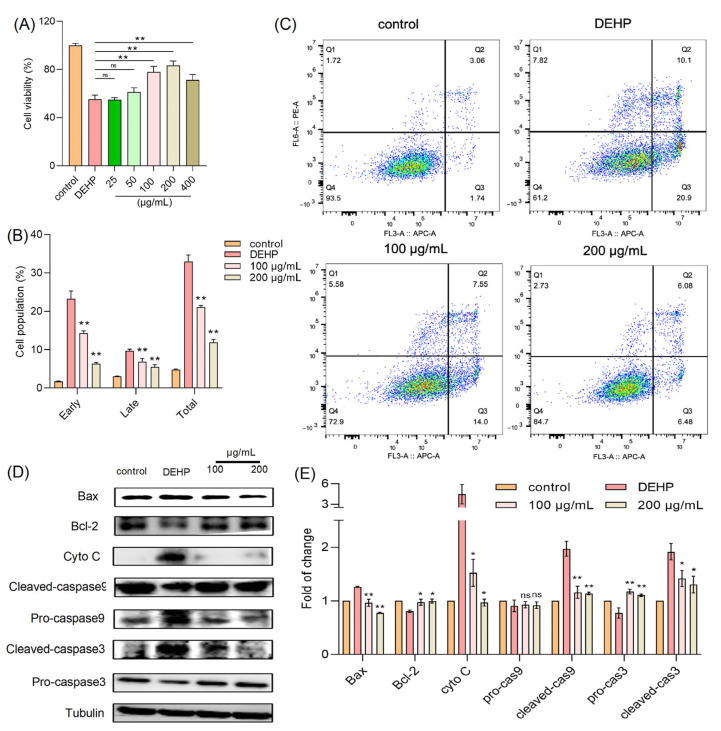
TFP reduces DEHP-induced cytotoxicity. (**A**) Cell viability was measured by MTT assay after cells were treated with TFP for 24 h and exposed to 2 mM DEHP for another 24 h. (**B**,**C**) Cell apoptosis was evaluated using AV/PI staining and analyzed via FlowJo™ Software v11 software. (**D**) Expression of apoptosis-related proteins was detected by SDS—PAGE and (**E**) the quantitative analysis of protein expression using Image J software. Data were analyzed by one-way ANOVA. “ns” indicates not significant; “*” indicates *p* < 0.05, and “**” indicates *p* < 0.01 compared with DEHP group.

Numerous studies have documented the cytotoxic effects of DEHP, a widely used plasticizer, on various cell types. To explore whether TFP can mitigate such effects, we employed an in vitro model using HUVECs. In our experiments, cells were treated with DEHP (2 mM) in the presence of varying concentrations of TFPs (25, 50, 100, 200, and 400 µg/mL), and the cell viability was assessed using the MTT assay. The results revealed that 2 mM DEHP alone reduced cell viability to approximately 55% of the control level (Figure 1A). Notably, the TFP treatment at 200 µg/mL significantly reversed this DEHP-induced cytotoxicity, restoring cell viability to around 84% (*p* < 0.01). Furthermore, the flow cytometry analysis of apoptosis showed that the DEHP treatment led to a 30% increase in apoptotic cells. However, the TFP treatment at concentrations of 100 and 200 µg/mL significantly reduced the apoptosis rate (*p* < 0.01) (Figure 1B,C).

These findings suggest that TFP can effectively alleviate DEHP-induced cytotoxicity in HUVECs, potentially through its protective and anti-apoptotic mechanisms. This evidence underscores the therapeutic potential of TFP in counteracting DEHP-associated cellular damage. Subsequently, we investigated the protein expression levels related to cell survival and apoptosis, including caspase-3, caspase-9, cytochrome c (cyto-c), Bax, and Bcl-2. Our results indicated that the DEHP treatment significantly increased the expression of apoptotic proteins such as cleaved caspase-3, cleaved caspase-9, cyto-c, and Bax, while markedly decreasing the expression of the anti-apoptotic protein Bcl-2 (Figure 1D,E). Conversely, the TFP treatment effectively suppressed the expression of these apoptosis-related proteins and enhanced the expression of survival-related proteins (Figure 1D,E). This further demonstrates that TFP can significantly counteract DEHP-induced cytotoxicity by modulating key proteins in the apoptotic pathway, thereby exerting a protective effect against DEHP-associated cellular damage.

### 3.2. TFP Restored the MMP and Reduced ROS in Cells

DEHP-induced cytotoxicity has been extensively studied, and growing evidence suggests that mitochondrial membrane potential (MMP) disruption and reactive oxygen species (ROS) generation are key mechanisms [21,22,23]. DEHP and its metabolites, such as mono(2-ethylhexyl) phthalate (MEHP), can induce oxidative stress, leading to mitochondrial dysfunction and ultimately triggering apoptosis.

**Figure 2:**
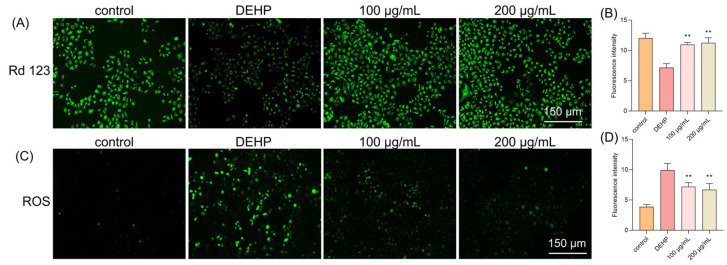
TFP restored the MMP and reduced ROS level in cells. The cells were exposed to DEHP (2 mM) for 24 h after they were treated with TFP for 24 h, and then (**A**) the MMP level was observed using Rd123 staining and (**B**) a quantitative analysis of the MMP; (**C**) the ROS level was observed under a fluorescence microscope and (**D**) the quantitative analysis of the ROS level. The fluorescence intensity in cells was analyzed using ImageJ software. All data were evaluated by one-way ANOVA. “**” indicates *p* < 0.01 compared with DEHP group.

In our study, we used Rhodamine 123 (Rh123), a cell membrane-permeable cationic fluorescent probe that is widely used to detect the MMP and monitor mitochondrial function in live cells. When cells undergo apoptosis or necrosis, MMP is lost and mitochondrial permeability transition pores open continuously, causing Rh123 to be released from mitochondria and leading to a significant decrease in mitochondrial fluorescence intensity. Our results showed that the DEHP treatment caused a marked decrease in fluorescence intensity, indicating a loss of MMP (Figure 2A). However, the TFP treatment significantly increased the fluorescence intensity, with 52.99% at 100 μg/mL and 56.76% at 200 μg/mL, suggesting that TFP may protect the MMP and prevent mitochondrial dysfunction (Figure 2B).

To clarify the role of ROS, we also used an ROS-specific fluorescent probe to observe intracellular ROS levels. The results showed that the DEHP treatment led to a significant increase in intracellular ROS, with 157.03% increasing compared with the control group (Figure 2C). While the TFP treatment markedly reduced ROS levels. At 100 μg/mL, the ROS level reduced by 27.54%, and a 32.41% reduction was observed when the concentration of TFP reached 200 μg/mL (Figure 2D). This indicates that DEHP-induced cytotoxicity may be mediated by ROS production, and TFP may alleviate this cytotoxicity by reducing ROS levels.

These findings suggest that DEHP-induced cytotoxicity is closely related to MMP disruption and ROS generation. TFP, through its antioxidant properties, may protect mitochondrial function and reduce ROS levels, thereby alleviating DEHP-induced cellular damage.

### 3.3. TFP Increased the Antioxidant Ability of Cells

**Figure 3:**
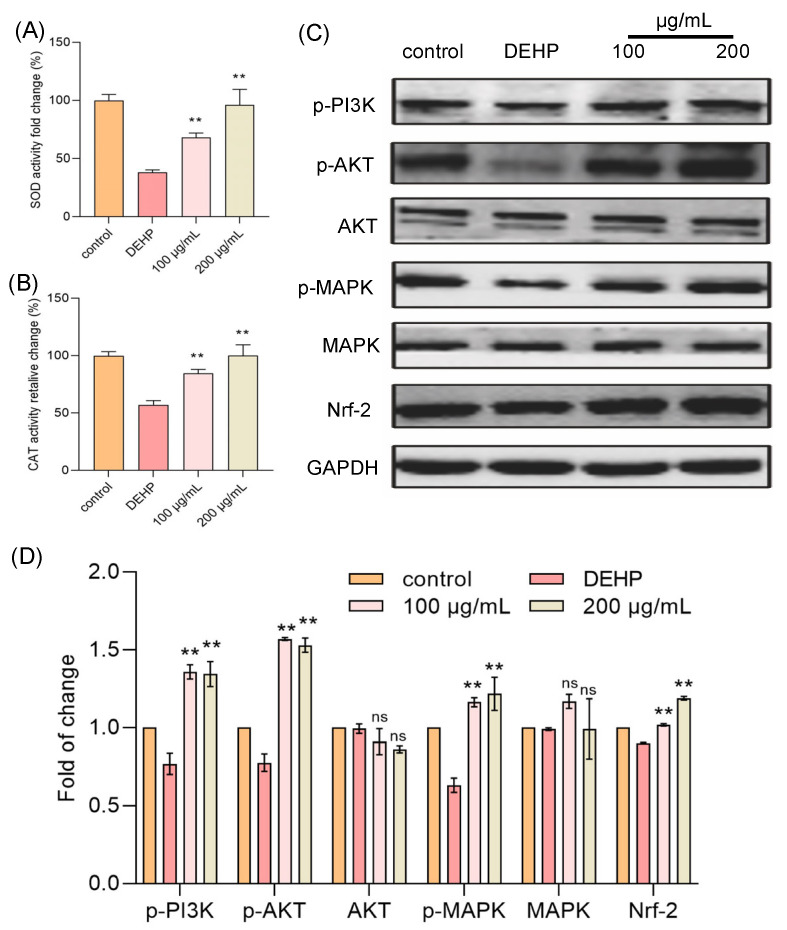
TFP activated MAPK/Nrf-2 pathway in cells. The cells were exposed to DEHP (2 mM) for 24 h after they were treated with TFP for 24 h, then the two main antioxidant enzymes were detected including (**A**) CAT and (**B**) SOD. (**C**) Expression analysis of antioxidant-related proteins via SDS—PAGE, and (**D**) the quantitative analysis of protein expression using Image J software. All data were analyzed by one-way ANOVA. “ns” indicates not significant; “**” indicates *p* < 0.01 compared with DEHP group.

To further clarify the mechanism by which TFP reduces intracellular ROS levels, we focused on two key antioxidant enzymes: superoxide dismutase (SOD) and catalase (CAT) [24]. SOD is a critical enzyme that dismutates superoxide radicals (O_2_^−^) into hydrogen peroxide (H_2_O_2_) and oxygen (O_2_), playing a pivotal role in the initial step of ROS scavenging. The generated H_2_O_2_ is then further decomposed into water and oxygen by CAT, which is a heme-containing enzyme with a high turnover rate. Both enzymes are indispensable components of the cellular antioxidant defense system, working in tandem to maintain redox balance and protect cells from oxidative damage. In our study, we aimed to determine whether TFP exerts its antioxidant effects by enhancing the activities of SOD and CAT. Cells were treated with DEHP and TFP, and the activities of these enzymes were measured. The results indicated that the DEHP treatment significantly reduced the activities of both SOD and CAT (Figure 3A,B). However, the TFP treatment markedly increased the activities of these enzymes. The activity of SOD increased 29.76% and 57.71% at 100 μg/mL and 200 μg/mL, respectively (Figure 3A). The activity of CAT was reduced to 57.02% when the cells were exposed to DEHP compared with the control group (Figure 3B). Interestingly, TFP significantly enhanced the activity of CAT by 27.45% and 42.98% at 100 μg/mL and 200 μg/mL, respectively (Figure 3B). This suggests that TFP may enhance the cell’s antioxidant capacity by upregulating SOD and CAT activities, thereby reducing ROS levels and alleviating DEHP-induced cytotoxicity.

### 3.4. TFP Activated MAPK/Nrf-2 Pathway

To further elucidate how TFP influences the activities of SOD and CAT to counteract DEHP-induced cytotoxicity, we looked into the relevant oxidative stress-related signaling pathways, the PI3K/AKT/MAPK and Nrf-2 pathways. The results showed that the DEHP treatment significantly reduced the expressions of PI3K, AKT, and MAPK, while the TFP treatment markedly increased their activities (Figure 3C,D). Furthermore, TFP upregulated the Nrf-2 expression that was downregulated by DEHP, leading to enhanced activities of SOD and CAT (Figure 3C,D). This indicates that TFP may enhance the cell’s antioxidant capacity by activating the PI3K/AKT/MAPK and Nrf-2 pathways, thereby reducing ROS levels and alleviating DEHP-induced cytotoxicity. Thus, our findings suggest that TFP exerts its protective effects against DEHP-induced cytotoxicity by modulating the PI3K/AKT/MAPK and Nrf-2 pathways, which in turn upregulate the activities of SOD and CAT.

### 3.5. TFP Protected C. elegans Under DEHP Condition

**Figure 4:**
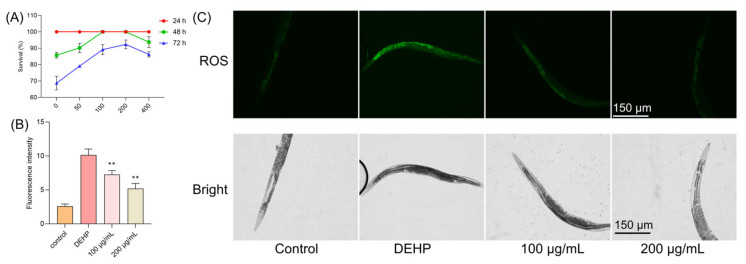
TFP protected *C. elegans* against DEHP-induced oxidative stress. (**A**) N2 worms were treated with different concentration of TFP from stages L1 to L4, and then the survival rate was recorded after being exposed to DEHP (40 mg/L) for different times, *n* > 60. (**B**,**C**) ROS levels in worms were visualized via fluorescence microscopy, with ROS fluorescence intensity quantified by a microplate reader. For the ROS assay, N2 worms received TFP treatment from stages L1 to L4, followed by 12 h DEHP exposure post-treatment, *n* > 60. All data underwent one-way ANOVA. “**” denotes *p* < 0.01 compared with DEHP group.

We further extended our investigation to an in vivo model using *Caenorhabditis elegans* to evaluate the protective effects of TFP against DEHP-induced toxicity. Nematodes were treated with different concentrations of TFP (100, 200, and 400 µg/mL) and exposed to DEHP for various durations. After 24 h of exposure, no significant changes in the survival rate of nematodes were observed. However, after 48 h of DEHP exposure, the survival rate of nematodes dropped to 85.74% (Figure 4A). Notably, the treatment with 100 and 200 µg/mL of TFP maintained the survival rate at 100% (Figure 4A). When the exposure time was extended to 72 h, the survival rate of nematodes treated with 100 µg/mL of TFP was 89.18%, and those treated with 200 µg/mL (Figure 4A) of TFP showed a survival rate of 92.3%, both significantly higher than the 68.75% survival rate of the control group (Figure 4A). Although the treatment with 400 µg/mL of TFP also improved survival rates, its effect was less pronounced than that of the 200 µg/mL concentration (Figure 4A). These results indicate that TFP can significantly enhance the survival rate of nematodes exposed to DEHP, with the most effective concentration being 200 µg/mL.

### 3.6. TFP Reduced the ROS in C. elegans Under DEHP Condition

DEHP exposure is known to elevate ROS levels in nematodes, adversely affecting their growth, development, and metabolism [25,26]. In our study, the DEHP treatment led to a significant increase in nematode ROS levels (Figure 4B). And a very high green fluorescence was observed when the worms were exposed to DEHP (Figure 4C). However, the TFP treatment resulted in a notable decrease in ROS levels (Figure 4B). At 100 µg/mL, the ROS level was reduced by 28.42%, and it was reduced by 48.92% at 200 µg/mL (Figure 4B), indicating that TFP can mitigate DEHP-induced oxidative damage in nematodes.

### 3.7. TFP Modulated Multiple Pathways to Regulate Daf-16

**Figure 5:**
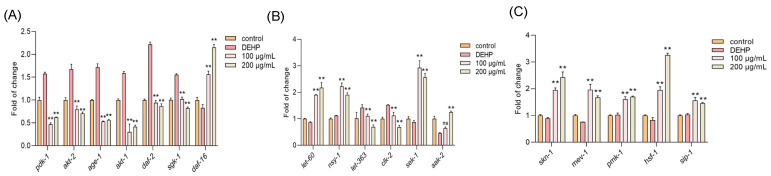
TFP activated *daf-16* and *skn-1* by modulating multiple pathways. N2 worms were treated with TFP from stages L1 to L4 before being exposed to DEHP (40 mg/L) for 12 h (*n* > 2000); the gene expression was detected, including (**A**) the gene expression of PI3K/AKT insulin signaling pathway, (**B**) the gene expression of mTOR and AMPK/MAPK pathways, and (**C**) the expressions of genes related to stress response system. All data underwent one-way ANOVA. “ns” denotes not significant; “**” denotes *p* < 0.01 compared with DEHP group.

Next, a quantitative real-time PCR (qPCR) was applied to explore the underlying mechanism that allows TFP to protect the nematodes against the DEHP toxicity. The results showed that DEHP exposure significantly upregulated the expression of PI3K/AKT insulin signaling pathway genes, including *pdk-1*, *akt-2*, *age-1*, *akt-1*, *daf-2*, and *sgk-1* (Figure 5A). However, the TFP treatment effectively downregulated these genes and concurrently activated the expression of *daf-16*, a pivotal gene in the insulin signaling pathway (Figure 5A). This suggests that TFP can suppress the PI3K/AKT insulin signaling pathway and enhance *daf-16* expression, which is consistent with previous studies by Brunet et al. and Padmanabhan et al. [27,28].

Additionally, DEHP exposure increased the expression of *let-363* and *clk-2*, which are associated with the mTOR pathway (Figure 5B). The TFP treatment attenuated this effect, indicating that TFP may modulate the mTOR pathway to support *daf-16* activation [29]. The TFP treatment also elevated the expression of *nsy-1*, *let-60*, *sek-1*, and *aak-2*, genes linked to the AMPK/MAPK pathway (Figure 5B). This upregulation implies that TFP can enhance AMPK/MAPK signaling to co-activate *daf-16* and boost the expression of its downstream target genes [30]. Moreover, TFP significantly increased the expression of *skn-1*, *mev-1*, *pmk-1*, *hsf-1*, and *sip-1*, components of the worms’ stress response system (Figure 5C). This indicates that TFP can strengthen the nematodes’ stress response, synergizing with *daf-16* to promote the expression of downstream stress target genes, as suggested by Oliveira et al. [31,32].

### 3.8. TFP Increased the Antioxidant System in Nematodes

**Figure 6:**
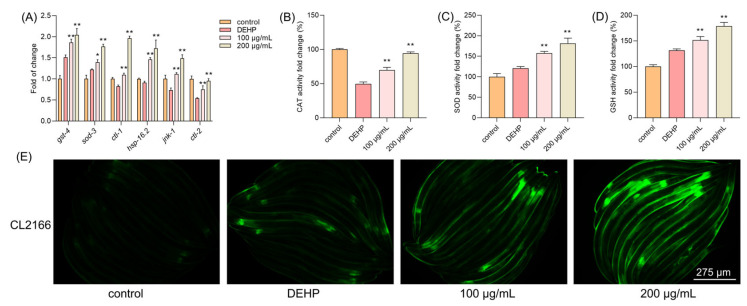
TFP increased the antioxidant ability in *C. elegans*. N2 worms were treated with TFP from stages L1 to L4 before being exposed to DEHP (40 mg/L) for 12 h (*n* > 2000); then (**A**) the gene expression involved in antioxidant system was detected, and three key antioxidant enzymes were assessed, including (**B**) CAT, (**C**) SOD, and (**D**) GSH. (**E**) The expression of GST-4::GFP in CL2166 strain was visualized using fluorescence microscopy after the worms were treated with TFP from stage L1 to L4 before being exposed to DEHP (40 mg/L) for 12 h. All data were analyzed using one-way ANOVA. “*” indicates *p* < 0.05, and “**” indicates *p* < 0.01 compared with DEHP group.

Since TFP can reduce the elevated levels of ROS induced by DEHP, we further explored the antioxidant system in *C. elegans*. The results demonstrated that the expressions of antioxidant genes were highly increased upon the TFP treatment, including *gst-4*, *sod-3*, *ctl-1*, *ctl-2*, *jnk-1*, and *hsp-16.2*, which are downstream of *daf-16* (Figure 6A). Moreover, the activity of CAT was increased by 20.12% and 44.70% at 100 µg/mL and 200 µg/mL, respectively (Figure 6B). Interestingly, DEHP exposure activated the expression of *sod-3* compared with the control group. Also, a high activity of SOD was detected in the DEHP group, which may contribute to the defense of *C. elegans* against the stress condition (Figure 6C). The TFP treatment increased the activity of SOD by 36.77% and 60.97% at 100 µg/mL and 200 µg/mL, respectively (Figure 6C). Meanwhile, a similar result was observed in GSH—that DEHP exposure activated the expression of gst-4 (Figure 6A) and the activity of GSH (Figure 6D). The activity of GSH was further increased by 19.95% and 47.33% at 100 µg/mL and 200 µg/mL, respectively (Figure 6D). Next, the CL2166 strain was utilized to visualize the expression of GST-4. Fluorescence microscopy revealed a significant increase in GST-4 expression following the TFP treatment, which aligns with the qPCR results (Figure 6E). This suggests that TFP may enhance the antioxidant system in *C. elegans*.

### 3.9. TFP Reduced the Expression of Cell Apoptosis Genes in C. elegans

**Figure 7:**
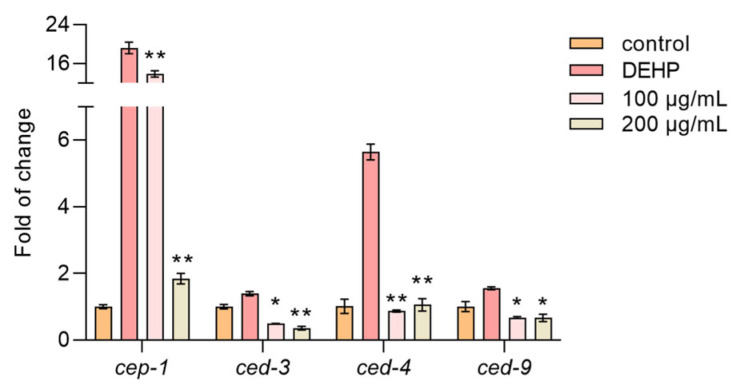
TFP downregulates the expression of apoptosis-associated genes. N2 worms were treated with TFP from stages L1 to L4 before being exposed to DEHP (40 mg/L) for 12 h (*n* > 2000), and then the expressions of genes involved in apoptosis were assessed. All data were analyzed using one-way ANOVA. “*” indicates *p* < 0.05, and “**” indicates *p* < 0.01 compared with DEHP group.

DEHP is known to induce apoptosis in nematode germline cells by upregulating apoptosis-related genes [33]. Our results showed that the DEHP treatment significantly increased the expression of *cep-1*, *ced-3*, *ced-4*, and *ced-9*, which are key genes involved in apoptosis. However, the TFP treatment markedly reduced the expression of these genes (Figure 7). This suggests that TFP can alleviate DEHP-induced cell apoptosis, aligning with our previous findings in cellular experiments where TFP reduced apoptosis rates. This evidence underscores TFP’s potential in mitigating DEHP-induced cytotoxicity and apoptosis in nematodes.

## 4. Discussion

DEHP is a widely used plasticizer, widely present in plastic products, food packaging, and cosmetics. Its long-term low-dose exposure through the food chain and respiratory tract has caused widespread health risks [26,34]. DEHP can interfere with the endocrine system of organisms through environmental exposure and is closely related to liver and kidney damage, reproductive toxicity, and metabolic disorders. Its core pathogenic mechanisms include inducing excessive ROS generation, triggering mitochondria-dependent apoptosis pathways, and inducing oxidative stress [35,36,37,38]. However, effective intervention measures for DEHP toxicity are still lacking. Natural polysaccharides have become a hot topic in current research due to their multi-target regulatory properties [39,40]. *Tremella fuciformis* is a medicinal and edible fungus, and its main component, TFP, has been shown to have antioxidant, anti-inflammatory, and immune regulatory activities, but its protective effect and molecular mechanism in DEHP-induced cross-species toxicity have not yet been clarified [12,41]. This study systematically revealed that TFP relieved DEHP toxicity in cell and nematode models by increasing the whole antioxidant ability, providing a theoretical basis for the development of environmental toxicological intervention agents based on natural polysaccharides.

At the cellular level, DEHP triggers a mitochondria-dependent apoptotic cascade by inducing the excessive production of ROS, which is manifested by the upregulation of the pro-apoptotic proteins Bax, caspase-3, caspase-9, and cytochrome C and the downregulation of the anti-apoptotic protein Bcl-2, ultimately leading to mitochondrial membrane potential collapse and cell apoptosis. This study found that TFP can significantly reverse the above process. On the one hand, DEHP inhibits the PI3K/AKT signaling pathway, leading to the upregulation of pro-apoptotic proteins Bax, caspase-3, caspase-9, and Cyto c, while TFP activated the anti-apoptotic pathway by increasing the expression of p-AKT and p-PI3K. This pathway can directly regulate AKT phosphorylation and inhibit pro-apoptotic proteins (such as BAD), block the release of mitochondrial apoptotic factors, and reduce caspase-3 activation, thereby blocking the execution stage of apoptosis [42,43,44]. This was consistent with the view of the “PI3K/AKT pathway as an anti-apoptotic hub” in the classical apoptosis regulation theory [45]. On the other hand, TFP removed excessive ROS by inducing Nrf-2 nuclear translocation and increasing the activity of its downstream antioxidant enzymes (CAT, SOD). At the same time, TFP further enhanced p-MAPK expression and synergistically promoted Nrf-2 nuclear translocation and the activity of antioxidant enzymes CAT and SOD, forming a cascade protection effect of “ROS removal—mitochondrial protection—apoptosis inhibition” [46,47,48]. It is worth noting that the recovery of the Bcl-2/Bax ratio and the maintenance of the mitochondrial membrane potential are mutually confirmed, indicating that TFP had dual interventions on the upstream signal (oxidative stress) and downstream execution stage (mitochondrial pathway) of apoptosis. This further proves the protective effect of TFP on mitochondria, avoiding mitochondrial dysfunction caused by oxidative damage. From the existing results, we can speculate that TFP may enhance the activity of CAT and SOD in cells by regulating the expression of Nrf-2, thereby reducing the high ROS levels induced by DEHP and thus reducing the occurrence of cell apoptosis. However, in this paper, only the expression of PI3K/AKT/MAPK was detected, and no corresponding inhibitors were used to confirm the involvement of these signaling pathways. Therefore, in future studies, more in-depth research should be carried out to prove the role of these signaling pathways in the protective effect of TFP.

In *C. elegans*, the protective mechanism of TFP shows more complex network regulation characteristics. TFP regulates the insulin signaling pathway (reducing the expression of *daf-2*, *age-1*, *akt-2*, etc.), relieves the inhibition of the longevity gene *daf-16* (FOXO family transcription factor) by the pathway, and directly activates *skn-1* (Nrf-2 homologous protein), thereby activating the antioxidant response of target genes (such as *gst-4*, *sod-3*, and *ctl-1*) [49,50,51,52]. This is similar to the activation of the Nrf-2/CAT/SOD pathway after the TFP treatment in cell experiments. At the same time, the activation of MAPK pathway-related genes (*pmk-1*, *sek-1*, and *nsy-1*) and heat shock factor *hsf-1* suggests that TFP may enhance the body’s overall stress resistance by enhancing the stress response pathway [53,54]. It is worth noting that the downregulation of TFP on nematode *cep-1* (p53 homologous gene) and *ced-3/-4* (apoptosis core gene) is highly consistent with the inhibitory effect of the caspase pathway in mammalian cells, indicating that its regulation of apoptosis core molecules is evolutionarily conservative. Therefore, in the nematode model, we can also infer that TFP can alleviate the oxidative damage caused by DEHP by regulating the body’s antioxidant capacity, which is highly conservative given the results in cell experiments. However, this study focused on exploring the mechanism of action of TFP in wild-type N2 and neglected to verify it with other corresponding mutants. Therefore, in future studies, corresponding mutants should be applied to explore whether the protective effect of TFP still exists, so as to determine the role of these genes.

Overall, this study, using cell and nematode models, revealed an underlying mechanism by which TFP mitigates DEHP toxicity. These findings provide a foundation for studying the potential of natural polysaccharides to mitigate damage induced by environmental toxicants. However, this study also has limitations. Although TFP demonstrated great protection against DEHP toxicity both in vitro and in vivo, the biotransformation mechanism of TFP within the complex metabolic enzyme system of mammals remains unclear. Therefore, further validation of TFP’s effects in mammals using a mouse model is warranted.

## 5. Conclusions

This study applied HUVECs and *C. elegans* to demonstrate the protection of TFP against DEHP-induced oxidative damage in vitro and in vivo. TFP reduced cell apoptosis and high ROS levels, which may contribute to increasing antioxidant enzymes through the PI3K/AKT/MAPK/Nrf-2 pathway. In *C. elegans*, TFP activated *daf-16/skn-1* by suppressing insulin-like pathways and upregulating the AMPK/MAPK pathway to enhance the antioxidant system, helping protect the worms against the oxidative stress induced by DEHP. These results offer a novel strategy for mitigating health risks posed by environmental pollutants like DEHP using natural compounds.

## Data Availability

The original contributions presented in the study are included in the article; further inquiries can be directed to the corresponding authors.

## Abbreviations

The following abbreviations are used in this manuscript:

CAT	Catalase
*C. elegans*	*Caenorhabditis elegans*
cyto-c	Cytochrome c
DAF-16	Abnormal Dauer Formation-16
DEHP	Di-(2-ethylhexyl) Phthalate
GSH	Glutathione
GST-4	Glutathione S-Transferase-4
HUVEC	Human Umbilical Vein Endothelial Cell
MAPK	Mitogen-Activated Protein Kinase
MEHP	Mono(2-ethylhexyl) Phthalate
MMP	Mitochondrial Membrane Potential
Nrf2	Nuclear Factor Erythroid 2-Related Factor 2
PPAR	Peroxisome Proliferator-Activated Receptor
ROS	Reactive Oxygen Species
SKN-1	Skinhead-1
SOD	Superoxide Dismutase
TFP	*Tremella fuciformis* Polysaccharides

## References

[1] Singh J., Jangra A., Kumar D. (2024). Recent advances in toxicological research of di-(2-ethylhexyl)-phthalate: Focus on endoplasmic reticulum stress pathway. Chemosphere.

[2] Giribabu N., Reddy P.S. (2017). Protection of male reproductive toxicity in rats exposed to di-n-butyl phthalate during embryonic development by testosterone. Biomed. Pharmacother..

[3] Dutta S., Sengupta P., Bagchi S., Chhikara B.S., Pavlík A., Sláma P., Roychoudhury S. (2023). Reproductive toxicity of combined effects of endocrine disruptors on human reproduction. Front. Cell Dev. Biol..

[4] Casals-Casas C., Desvergne B. (2017). Endocrine disruptors: From endocrine to metabolic disruption. Annu. Rev. Physiol..

[5] Schettler T. (2006). Human exposure to phthalates via consumer products. Int. J. Androl..

[6] Pogrmic-Majkic K., Nenadov D.S., Tesic B., Nedeljkovic S.F., Kokai D., Stanic B., Andric N. (2022). Mapping DEHP to the adverse outcome pathway network for human female reproductive toxicity. Arch. Toxicol..

[7] Liu Y., Guo Z., Zhu R., Gou D., Jia P.-P., Pei D.-S. (2023). An insight into sex-specific neurotoxicity and molecular mechanisms of DEHP: A critical review. Environ. Pollut..

[8] Lin C.-H., Chen T.-J., Chen S.-S., Hsiao P.-C., Yang R.-C. (2011). Activation of Trim17 by PPARγ is involved in Di(2-ethylhexyl) phthalate (DEHP)-induced apoptosis on Neuro-2a cells. Toxicol. Lett..

[9] Schaedlich K., Gebauer S., Hunger L., Beier L.-S., Koch H.M., Wabitsch M., Fischer B., Ernst J. (2018). DEHP deregulates adipokine levels and impairs fatty acid storage in human SGBS-adipocytes. Sci. Rep..

[10] Ashaari S., Jamialahmadi T., Davies N.M., Almahmeed W., Sahebkar A. (2025). Di (2-ethyl hexyl) phthalate and its metabolite-induced metabolic syndrome: A review of molecular mechanisms. Drug Chem. Toxicol..

[11] Liu T.-T., Hong K.-S., Yang T.-S. (2024). Functionalities of *Tremella fuciformis* polysaccharides modified with gallic acid. Molecules.

[12] Ma X., Yang M., He Y., Zhai C., Li C. (2021). A review on the production, structure, bioactivities and applications of Tremella polysaccharides. Int. J. Immunopathol. Pharmacol..

[13] Lin Y., Xu Q., Li X., Shao P. (2022). *Tremella fuciformis* polysaccharides as a fat substitute on the rheological, texture and sensory attributes of low-fat yogurt. Curr. Res. Food Sci..

[14] Zhuang W., Zheng S., Chen F., Gao S., Zhong M., Zheng B. (2023). Effects of *Tremella fuciformis* mushroom polysaccharides on structure, pasting, and thermal properties of Chinese Chestnuts (*Castanea henryi*) starch granules under different freeze-thaw cycles. Foods.

[15] Wu Q., Zheng C., Ning Z.-X., Yang B. (2007). Modification of low molecular weight polysaccharides from *Tremella fuciformis* and their antioxidant activity in vitro. Int. J. Mol. Sci..

[16] Bin C. (2010). Optimization of extraction of *Tremella fuciformis* polysaccharides and its antioxidant and antitumour activities in vitro. Carbohydr. Polym..

[17] Gitsomboon S., Ratanapornsompong G., Ongphiphadhanakul B., Thongpradit S., Chanprasertyothin S., Chailurtkit L.-O., Nimitphong H. (2024). *Tremella fuciformis* beverage improves glycated hemoglobin A1c and waist circumference in overweight/obese prediabetic subjects: A randomized controlled trial. BMC Nutr..

[18] Bach E., Costa S.G., Oliveira H.A.d., Junior J.A.S., Silva K.M.d., Marco R.M.d., Hi E.M.B., Wadt N.S.Y. (2015). Use of polysaccharide extracted from *Tremella fuciformis* Berk for control diabetes induced in rats. Emir. J. Food Agric..

[19] Cao Y. (2025). Human Umbilical Vein Endothelial Cells (HUVECs) in Pharmacology and Toxicology: A Review. J. Appl. Toxicol..

[20] Hunt P.R. (2017). The *C. elegans* model in toxicity testing. J. Appl. Toxicol..

[21] Pandey V., Sharma A., Tiwari S., Patel Y., Chauhan J.K., Ayesha S., Sahu A.N., Gupta R., Tripathi A., Dubey P.K. (2024). Shatavarin-IV rescues the Di (2-ethylhexyl) phthalate (DEHP) induced oxidative stress in rat granulosa cells in vitro. Reprod. Toxicol..

[22] Cai H., Li K., Yin Y., Ni X., Xu S. (2023). Quercetin alleviates DEHP exposure-induced pyroptosis and cytokine expression changes in grass carp L8824 cell line by inhibiting ROS/MAPK/NF-κB pathway. Fish Shellfish Immunol..

[23] Guo Y., Li B., Yan Y., Zhang N., Shao S., Yang L., Ouyang L., Wu P., Ma F., Duan H. (2025). Di-(2-ethylhexyl) phthalate exposure induces developmental toxicity in the mouse fetal heart via mitochondrial dysfunction. Cardiovasc. Toxicol..

[24] Drevet J.R. (2006). The antioxidant glutathione peroxidase family and spermatozoa: A complex story. Mol. Cell. Endocrinol..

[25] Tseng I.-L., Yang Y.-F., Yu C.-W., Li W.-H., Liao V.H.-C. (2013). Phthalates induce neurotoxicity affecting locomotor and thermotactic behaviors and AFD neurons through oxidative stress in *Caenorhabditis elegans*. PLoS ONE.

[26] Li J., Qu M., Wang M., Yue Y., Chen Z., Liu R., Bu Y., Li Y. (2021). Reproductive toxicity and underlying mechanisms of di(2-ethylhexyl) phthalate in nematode *Caenorhabditis elegans*. J. Environ. Sci..

[27] Brunet A., Bonni A., Zigmond M.J., Lin M.Z., Juo P., Hu L.S., Anderson M.J., Arden K.C., Blenis J., Greenberg M.E. (1999). Akt promotes cell survival by phosphorylating and inhibiting a Forkhead transcription factor. Cell.

[28] Padmanabhan S., Mukhopadhyay A., Narasimhan S.D., Tesz G., Czech M.P., Tissenbaum H.A. (2009). A PP2A regulatory subunit regulates *C. elegans* insulin/IGF-1 signaling by modulating AKT-1 phosphorylation. Cell.

[29] Jia K., Chen D., Riddle D.L. (2004). The TOR pathway interacts with the insulin signaling pathway to regulate *C. elegans* larval development, metabolism and life span. Development.

[30] Greer E.L., Dowlatshahi D., Banko M.R., Villen J., Hoang K., Blanchard D., Gygi S.P., Brunet A. (2007). An AMPK-FOXO pathway mediates longevity induced by a novel method of dietary restriction in *C. elegans*. Curr. Biol..

[31] An J.H., Blackwell T.K. (2003). SKN-1 links *C. elegans* mesendodermal specification to a conserved oxidative stress response. Genes Dev..

[32] Oliveira R.P., Abate J.P., Dilks K., Landis J., Ashraf J., Murphy C.T., Blackwell T.K. (2009). Condition-adapted stress and longevity gene regulation by *Caenorhabditis elegans* SKN-1/Nrf. Aging Cell.

[33] Yin J., Liu R., Jian Z., Yang D., Pu Y., Yin L., Wang D. (2018). Di (2-ethylhexyl) phthalate-induced reproductive toxicity involved in dna damage-dependent oocyte apoptosis and oxidative stress in *Caenorhabditis elegans*. Ecotoxicol. Environ. Saf..

[34] Braver-Sewradj S.P.D., Piersma A., Hessel E.V.S. (2020). An update on the hazard of and exposure to diethyl hexyl phthalate (DEHP) alternatives used in medical devices. Crit. Rev. Toxicol..

[35] Caldwell J.C. (2012). DEHP: Genotoxicity and potential carcinogenic mechanisms—A review. Mutat. Res..

[36] Rowdhwal S.S.S., Chen J. (2018). Toxic effects of Di-2-ethylhexyl phthalate: An overview. BioMed Res. Int..

[37] Yi W.E.I., Xiang-Liang T., Yu Z., Bin L., Lian-Ju S., Chun-lan L., Tao L.I.N., Da-wei H.E., Sheng-de W.U., Guang-hui W.E.I. (2018). DEHP exposure destroys blood-testis barrier (BTB) integrity of immature testes through excessive ROS-mediated autophagy. Genes Dis..

[38] Tripathi A., Pandey V., Sahu A.N., Singh A., Dubey P.K. (2019). Di-(2-ethylhexyl) phthalate (DEHP) inhibits steroidogenesis and induces mitochondria-ROS mediated apoptosis in rat ovarian granulosa cells. Toxicol. Res..

[39] Schepetkin I.A., Quinn M.T. (2006). Botanical polysaccharides: Macrophage immunomodulation and therapeutic potential. Int. Immunopharmacol..

[40] Cai Y., Guo J., Kang Y. (2024). Future prospect of polysaccharide as a potential therapy in hepatocellular carcinoma: A review. Int. J. Biol. Macromol..

[41] Yuan H., Dong L., Zhang Z., He Y., Ma X. (2022). Production, structure, and bioactivity of polysaccharide isolated from *Tremella fuciformis*. Food Sci. Hum. Wellness.

[42] Lee E.-R., Kim J.-Y., Kang Y.-J., Ahn J.-Y., Kim J.-H., Kim B.-W., Choi H.-Y., Jeong M.-Y., Cho S.-G. (2006). Interplay between PI3K/Akt and MAPK signaling pathways in DNA-damaging drug-induced apoptosis. Biochim. Biophys. Acta.

[43] Kulik G., Klippel A., Weber M.J. (1997). Antiapoptotic signalling by the insulin-like growth factor I receptor, phosphatidylinositol 3-kinase, and Akt. Mol. Cell. Biol..

[44] Dudek H., Datta S.R., Franke T.F., Birnbaum M.J., Yao R., Cooper G.M., Segal R.A., Kaplan D.R., Greenberg M.E. (1997). Regulation of neuronal survival by the serine-threonine protein kinase Akt. Science.

[45] Kennedy S.G., Wagner A.J., Conzen S.D., Jordán J., Bellacosa A., Tsichlis P.N., Hay N. (1997). The PI 3-kinase/Akt signaling pathway delivers an anti-apoptotic signal. Genes Dev..

[46] Vomhof-Dekrey E.E., Picklo M.J. (2012). The Nrf2-antioxidant response element pathway: A target for regulating energy metabolism. J. Nutr. Biochem..

[47] Nguyen T., Nioi P., Pickett C.B. (2009). The Nrf2-antioxidant response element signaling pathway and its activation by oxidative stress. J. Biol. Chem..

[48] Li W., Kong A.-N. (2009). Molecular mechanisms of Nrf2-mediated antioxidant response. Mol. Carcinog..

[49] Tullet J.M.A. (2015). DAF-16 target identification in *C. elegans*: Past, present and future. Biogerontology.

[50] Park S.-K., Tedesco P.M., Johnson T.E. (2009). Oxidative stress and longevity in *Caenorhabditis elegans* as mediated by SKN-1. Aging Cell.

[51] Tullet J.M.A., Green J.W., Au C., Benedetto A., Thompson M.A., Clark E., Gilliat A.F., Young A., Schmeisser K., Gems D. (2017). The SKN-1/Nrf2 transcription factor can protect against oxidative stress and increase lifespan in *C. elegans* by distinct mechanisms. Aging Cell.

[52] Sun X., Chen W.-D., Wang Y.-D. (2017). DAF-16/FOXO transcription factor in aging and longevity. Front. Pharmacol..

[53] Inoue H., Hisamoto N., An J.H., Oliveira R.P., Nishida E., Blackwell T.K., Matsumoto K. (2005). The *C. elegans* p38 MAPK pathway regulates nuclear localization of the transcription factor SKN-1 in oxidative stress response. Genes Dev..

[54] Brunquell J., Morris S., Lu Y., Cheng F., Westerheide S.D. (2016). The genome-wide role of HSF-1 in the regulation of gene expression in *Caenorhabditis elegans*. BMC Genom..

